# An autocrine/paracrine circuit of growth differentiation factor (GDF) 15 has a role for maintenance of breast cancer stem-like cells

**DOI:** 10.18632/oncotarget.15276

**Published:** 2017-02-11

**Authors:** Asako Sasahara, Kana Tominaga, Tatsunori Nishimura, Masao Yano, Etsuko Kiyokawa, Miki Noguchi, Masakuni Noguchi, Hajime Kanauchi, Toshihisa Ogawa, Hiroshi Minato, Keiichiro Tada, Yasuyuki Seto, Arinobu Tojo, Noriko Gotoh

**Affiliations:** ^1^ Division of Molecular Therapy, Institute of Medical Science, University of Tokyo, Tokyo, Japan; ^2^ Department of Breast and Endocrine Surgery, Graduate School of Medicine, University of Tokyo, Tokyo, Japan; ^3^ Division of Cancer Cell Biology, Cancer Research Institute, Kanazawa University, Kanazawa, Japan; ^4^ Department of Surgery, Minamimachida Hospital, Machida, Tokyo, Japan; ^5^ Department of Oncologic Pathology, Kanazawa Medical University, Ishikawa, Japan; ^6^ Department of General and Digestive Surgery, Kanazawa Medical University, Ishikawa, Japan; ^7^ Department of Breast and Endocrine Surgery, Showa General Hospital, Kodaira, Tokyo, Japan; ^8^ Department of Pathology and Laboratory Medicine, Kanazawa Medical University, Ishikawa, Japan; ^9^ Department of Gastrointestinal Surgery, Graduate School of Medicine, University of Tokyo, Tokyo, Japan

**Keywords:** tumor spheres, cancer stem cells, breast cancer, TGFbeta, ERK

## Abstract

Cancer stem cells are thought to be responsible for tumor growth, recurrence, and resistance to conventional cancer therapy. However, it is still unclear how they are maintained in tumor tissues. Here, we show that the growth differentiation factor 15 (GDF15), a member of the TGFβ family, may maintain cancer stem-like cells in breast cancer tissues by inducing its own expression in an autocrine/paracrine manner. We found that GDF15, but not TGFβ, increased tumor sphere formation in several breast cancer cell lines and patient-derived primary breast cancer cells. As expected, TGFβ strongly stimulated the phosphorylation of Smad2. GDF15 also stimulated the phosphorylation of Smad2, but the GDF15-induced tumor sphere forming efficiency was not significantly affected by treatment with SB431542, an inhibitor of the TGFβ signaling. Although TGFβ transiently activated ERK1/2, GDF15 induced prolonged activation of ERK1/2. Treatment with U0126, an inhibitor of the MEK-ERK1/2 signaling, greatly inhibited the GDF15-induced tumor sphere formation. Moreover, cytokine array experiments revealed that GDF15, but not TGFβ, is able to induce its own expression; furthermore, it appears to form an autocrine/paracrine circuit to continuously produce GDF15. In addition, we found heterogeneous expression levels of GDF15 among cancer cells and in human breast cancer tissues using immunohistochemistry. This may reflect a heterogeneous cancer cell population, including cancer stem-like cells and other cancer cells. Our findings suggest that GDF15 induces tumor sphere formation through GDF15-ERK1/2-GDF15 circuits, leading to maintenance of GDF15^high^ cancer stem-like cells. Targeting GDF15 to break these circuits should contribute to the eradication of tumors.

## INTRODUCTION

Breast cancer is the most common type of cancer and a leading cause of death among women around the world [1]. The subtypes of breast cancer are classified clinically as luminal A, luminal B, luminal B-like HER2 negative, luminal B-like HER2-positive, HER2 type, and triple negative, based on the patterns of immunohistochemical staining in the tumor tissue. The luminal A and luminal B subtypes are positive for hormone receptors, estrogen receptors (ER-positive) and/or progesterone receptors (PgR-positive); the luminal B-like HER2-negative subtype shows a higher Ki67 index, which indicates a high proliferative capacity, and luminal B-like HER2-positive subtype also stains positie for HER2. The triple negative subtype stains negative for the hormone receptors and is HER2-negative. Recent advancements in medical technology have developed various treatment options for breast cancer, such as targeted therapies against the hormone receptors and HER2. Although a substantial number of patients respond well to this approach, metastases and recurrence continue to occur [2]. Recent emerging evidence suggests that cancer stem cells (CSCs) are responsible for tumor growth, recurrence, and resistance to conventional cancer therapy [3]; therefore, target therapies against CSCs are needed. Tumor tissues are comprised of very heterogeneous cell types and are thought to be in a hierarchical organization that includes CSCs and their progenies, similar to how normal tissue is derived from tissue-specific stem cells [3–7]. Because it is still mostly unclear how CSCs are maintained in tumor tissues, revealing the mechanism of CSC maintenance is an urgent need in order to establish a CSCs targeted therapy. CSCs represent a distinct cell population with the capacity for self-renewal. Cancer cells that exhibit some CSC properties have been detected in many solid tumors, including breast cancer [5, 8]. The ability for *in vitro* tumor sphere formation has been established as a property of CSCs [9, 10]. Tumor spheres are floating cell aggregates that are produced when cancer cells are cultured in a defined sphere culture medium (SCM) containing a cocktail of growth factors and hormones. Epithelial cells do not survive in suspension, however, cells with stem-like properties are thought to survive and be able to divide in suspension. We have previously reported that heregulin or IGF2 is able to induce tumor sphere formation as a single cytokine [11, 12]. Because this is a good indication that they play critical roles for maintenance of cancer stem-like cells, it is important to examine if there are other cytokines that have similar activity.

The TGFβ family is a group of cytokines with pleiotropic functions [13–15]. This family has 33 members, including TGFβ and growth differentiation factors (GDFs). They are involved in the regulation of various biological functions such as proliferation, migration, differentiation, and apoptosis in many different cell types. The binding of TGFβ-family proteins to cell-surface receptor complexes enables the TGFβ type II receptor kinases to phosphorylate, and thus activate, TGFβ type I receptor kinases, which then phosphorylate the intracellular signaling proteins, Smad2/3. Once phosphorylated, the Smad2/3 complex binds to Smad4 and becomes activated; the complex can then translocate to the nucleus and initiate transcription. The Smad pathway for gene regulation is the canonical pathway of the TGFβ family [14, 16]. TGFβ signaling regulates the expression of various genes in a highly context-dependent manner, which is mediated by complex interactions between Smads and other signaling pathways [13, 15].

GDF15, also known as MIC-1, PTGF-β, PDF, PLAB, PL74, and NAG-1, is a divergent member of the TGF-β family [17–19]. Under normal conditions, the only tissue that expresses large amounts of GDF15 is the placenta. GDF15 is elevated in various cell types, including macrophages, epithelial cells, and fibroblasts in response to acute injury, inflammation, and malignancy [17, 20]. The role of GDF15 is broad. In cancer, it is reported that elevated serum levels of GDF15 cause cancer-induced anorexia and cachexia directly through circulating GDF15 on feeding centers in the brain [21]. Although several studies reported that GDF15 functions as a tumor suppressor by arresting the cell cycle and leading to apoptosis [17, 22–24], there are numerous reports stating that GDF15 has a pro-tumorigenic ability [17, 22, 23, 25]. Other studies showed that GDF15 can be a biomarker of poor prognosis in both serum and cancer tissues [23]. On the other hand, it is still largely unknown whether GDF15 has any roles in CSCs from a vast majority of tumors, including breast cancer. Moreover, it is largely unclear the signaling pathways by which GDF15 exerts its biological functions.

In this study, we showed that GDF15 induces tumor sphere formation, an important property of CSCs, in breast cancer cells using patient-derived primary breast cancer cells. We also showed that GDF15 induces its own expression in breast cancer cells through sustained activation of ERK1/2. This GDF15-ERK1/2-GDF15 circuit may maintain cancer stem-like cells in an autocrine/paracrine manner. Finally, we showed that expression levels of GDF15 are heterogeneous among cancer cells from human breast cancer tissue samples. Cells with high levels of GDF15 may maintain GDF15 production by the autocrine/paracrine circuit and act as cancer stem-like cells in breast cancer. Therapies targeted against GDF15, such as anti-GDF15 antibodies, would be useful for eradication of GDF15^high^ cancer stem-like cells.

## RESULTS

### GDF15, but not TGFβ, efficiently induces tumor sphere formation in breast cancer cells

**Figure 1:**
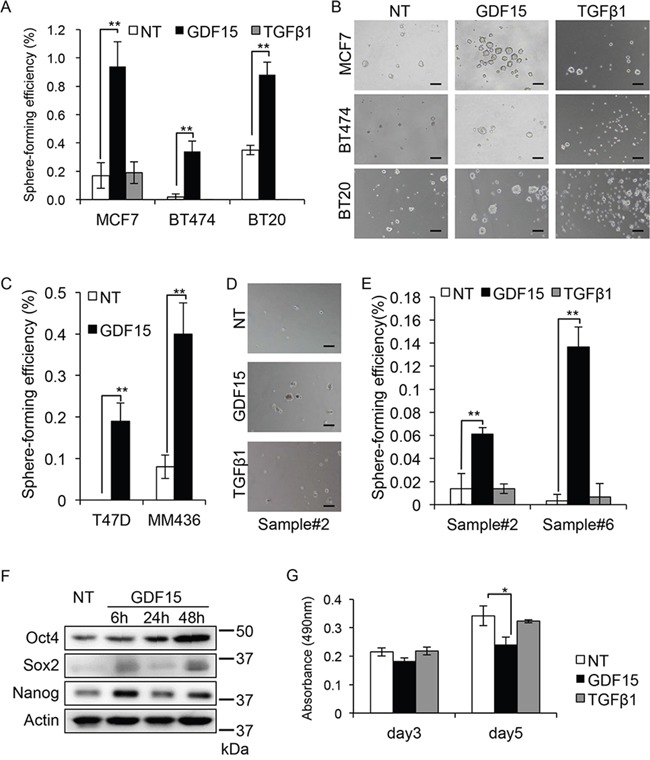
GDF15, but not TGFβ, efficiently induces tumor sphere formation in breast cancer cells **A**. Tumor sphere assay of cell lines treated with GDF15 (200 ng/mL) or TGFβ1 (200 ng/mL). NT, not treated. n=4. **P < 0.01. **B**. Representative images of tumor spheres observed in (A). NT, not treated. Scale bar: 100 μm. **C**. Tumor sphere assay of cell lines treated with GDF15 (200 ng/mL). NT, not treated. n = 4, **P < 0.01. **D**. Representative images of tumor spheres derived from clinical sample #2, treated with GDF15 (200 ng/mL) or TGFβ1 (200 ng/mL). NT, not treated. Scale bar: 100 μm. **E**. The number of spheres in (D) and clinical sample #6 was counted and the percentage of sphere-forming efficiency was recorded. NT, not treated. n=4. **P < 0.01. **F**. Immunoblotting analysis of Oct4, Sox2, and Nanog expression in MCF7 cells treated with GDF15 (200 ng/mL). NT, not treated. Actin was used as a loading control. **G**. MTT assay using MCF7 cells. Cells were seeded to a 96-well plate and starved overnight prior to treatment with GDF15 (200 ng/mL) or TGFβ1 (200 ng/mL). The data was recorded at the indicated time. n=3. *P < 0.05.

Tumor sphere forming ability is an important property of cancer stem-like cells. To investigate the possibility that GDF15 has any functions in CSCs, we performed tumor sphere formation assay [11]. We found that GDF15 strongly induced tumor sphere formation in the luminal type MCF7, HER2-positive type BT474, and basal type BT20 cells, which are cell lines representative of each subtype of breast cancer (Figure 1A and 1B). By contrast, we found that TGFβ did not induce the formation of tumor spheres in BT474 and BT20 cells. TGFβ did not strongly induce tumor sphere formation in the MCF7 cells compared with that in un-treated control MCF7 cells. We, therefore, focused on the ability of GDF15 to form tumor spheres, and examined the additional cell lines, T47D and MDA-MB-436 (MM436), which represent the luminal and basal subtype, respectively. As expected, the sphere-forming efficiency in both cell lines was significantly increased by stimulation with GDF15 (Figure 1C).

Because cancer cell lines are artificially immortalized, they may have limited usefulness for analyzing tumor sphere forming ability. It is thus important to use early-passage patient-derived primary cancer cells. We next examined the tumor sphere forming ability of patient-derived primary breast cancer cells. We analyzed 8 clinical samples from which tumor spheres were formed in SCM. Among them, GDF15, but not TGFβ, clearly induced tumor spheres (2/8 samples) (Figure 1D and 1E and Supplementary Table 1).

We chose to use the MCF7 breast cancer cells because they show a good response to GDF15 (Figure 1A). We then examined another property of cancer stem-like cells, which is the expression of stem-cell markers Oct4, Sox2, and Nanog, after stimulation with GDF15 [26]. We found that the levels of expression of the three markers were increased after 6 and 50 hours of stimulation with GDF15 (Figure 1F). This result suggests that GDF15 induces stem cell-like properties in breast cancer cells.

We next examined the effect of GDF15 on cell proliferation and confirmed that GDF15 does not have significant positive effects on cell proliferation; on the contrary, it even reduced cell proliferation in the MCF7 cells (Figure 1G).

### Activation of the canonical Smad pathway is not required for the GDF15-induced tumor sphere formation

**Figure 2:**
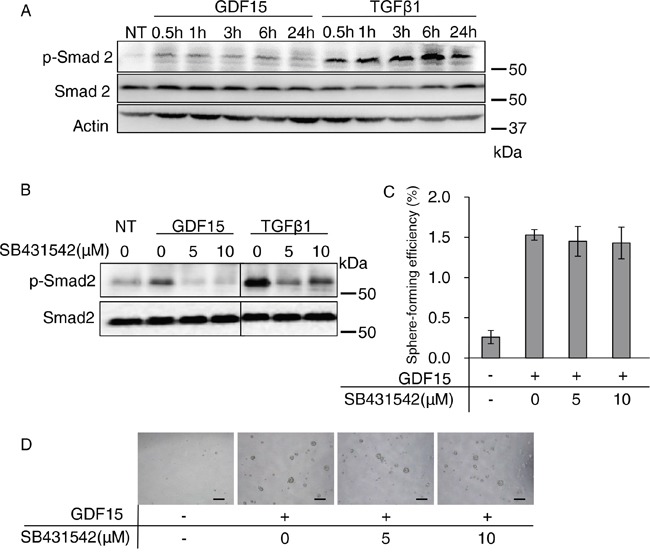
Activation of the canonical Smad pathway is not required for GDF15-induced tumor sphere formation **A**. Immunoblotting analysis of phosphorylated Smad2 (p-Smad2) and Smad2 expression in MCF7 cells treated with GDF15 (200 ng/mL) or TGFβ1 (200 ng/mL). NT, not treated. Actin was used as a loading control. **B**. Immunoblotting analysis of p-Smad2 and Smad2 expression in MCF7 cells. An indicated concentration of SB431542 was applied to the cells 60 minutes prior to treatment with GDF15 (200 ng/mL) or TGFβ1 (200 ng/mL). Cell lysates were collected 30 minutes after treatment with each ligand. NT, not treated. **C**. Tumor sphere assay of GDF15-treated (200 ng/mL) MCF7 cells in presence of the indicated concentrations of SB431542. n=4. **D**. Representative images of (C). Scale bar: 100 μm.

Although several studies reported the activation of Smads [21], or ERK1/2 [27–29], in GDF15 signaling, the results are controversial. To analyze the GDF15-induced signaling pathways involved in tumor sphere formation, we first examined the phosphorylation of Smad2 by western blot analysis to determine whether GDF15 stimulates the Smad pathways in MCF7 breast cancer cells. As expected, TGFβ strongly phosphorylates Smad2 (Figure 2A). GDF15 modestly stimulated phosphorylation of Smad2.

To examine whether signaling through the TGFβ type I receptor is required for tumor sphere formation, we treated cells with SB431542, a selective inhibitor of TGFβ type I receptor kinases that are ALK4, ALK5, and ALK7 [14]. We confirmed that the GDF15- or TGFβ-stimulated phosphorylation of Smad2 was inhibited by SB431542 (Figure 2B). However, the GDF15-induced tumor sphere formation was not significantly affected by treatment with SB431542 (Figure 2C and 2D). These results indicate that the GDF15-induced tumor sphere formation does not require strong activation of the canonical Smad2/4 pathway.

### Prolonged activation of ERK1/2 appears to be required for GDF15-induced tumor sphere formation

**Figure 3:**
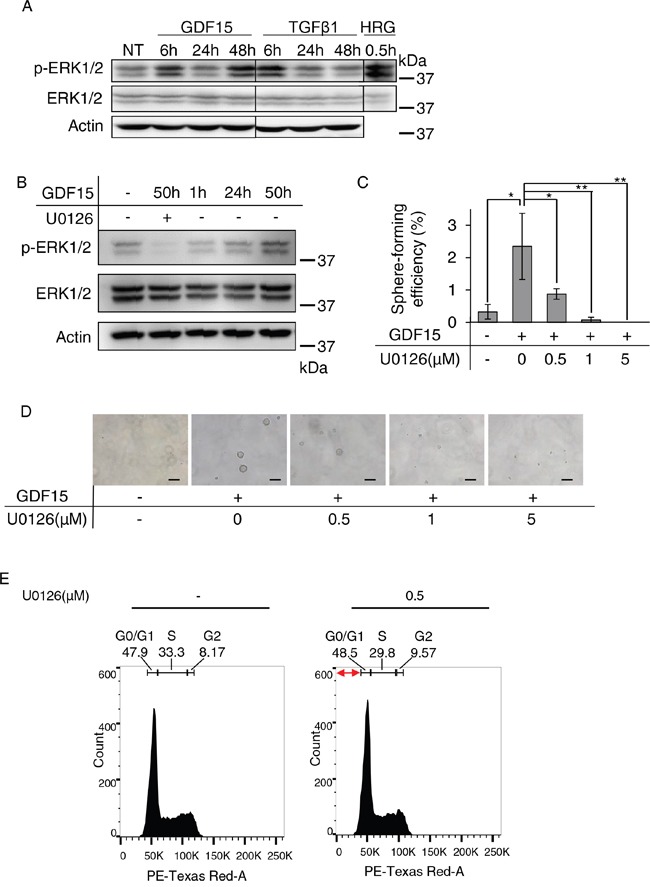
Sustained activation of ERK1/2 appears to be required for GDF15-induced tumor sphere formation **A**. Immunoblotting analysis of phosphorylated ERK1/2 (p-ERK1/2) and ERK1/2 expression in MCF7 cells treated with GDF15 (200 ng/mL) or TGFβ1 (200 ng/mL). NT, not treated. Actin was used as a loading control. The lysate of MCF7 cells stimulated with heregulin (HRG) was used as a positive control. **B**. Immunoblotting analysis of p-ERK1/2 and ERK1/2 expression in MCF7 cells treated with GDF15 (200 ng/mL) in the presence or absence of U0126 (5 μM). **C**. Sphere formation assay of GDF15-treated (200 ng/mL) MCF7 cells in the presence or absence of the indicated concentration of U0126. n=4. **P < 0.01, *P < 0.05. **D**. Representative images of (C). Scale bar: 100 μm. **E**. Cell cycle analysis of MCF7 cells treated with GDF15 (200 ng/mL) in the presence or absence of U0126 (0.5 μM). Apoptotic cells are observed in the region indicated with the red arrows (the sub-G1 area).

We next examined the phosphorylation of ERK1/2 by western blot analysis to determine whether GDF15 stimulates their activation. We found that 48 hours post-treatment, GDF15, but not TGFβ1, stimulated strong phosphorylation of ERK1/2 over an extended period of time (Figure 3A). To examine whether activation of ERK1/2 is required for tumor sphere formation, we treated cells with U0126, a selective inhibitor for MEK. We confirmed that the GDF15-stimulated phosphorylation of ERK1/2 was inhibited by treatment with U0126 for 50 hours after stimulation (Figure 3B). We found that the GDF15-induced tumor sphere formation was inhibited by treatment with U0126 at a low dose (0.5 μM) and in a dose-dependent manner (Figure 3C and 3D).

We analyzed the phases of the cell cycle using cells stimulated with GDF15 in the presence, or absence, of U0126. After 50 hours of stimulation with GDF15, the ratios of G0/G1, S, and G2 phases were not significantly different between cells cultured in the presence of U0126 and those cultured it its absence (Figure 3E). There were few apoptotic cells in sub-G1 area, and there was no difference between the cells cultured in the presence of U0126 and those cultured in its absence. We, thus, confirmed that treatment with U0126 does not significantly inhibit cell proliferation or induce apoptosis under these conditions. These results suggest that activation of ERK1/2 over a prolonged period of time is required for GDF15-induced tumor sphere formation.

### GDF15 induces its own expression in a delayed time course

**Figure 4:**
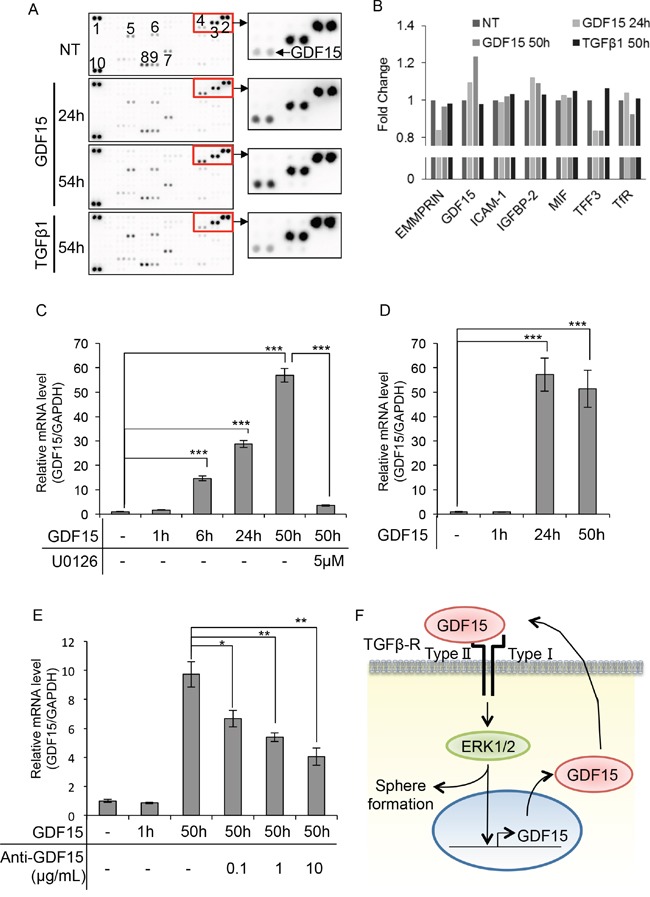
GDF15 induces its own expression in a delayed time course **A**. Cytokine array of MCF7 cells treated with GDF15 (200 ng/mL) or TGFβ1 (200 ng/mL). NT, not treated. The numbers of spots indicate cytokines: 1, 2, and 10, reference spots; 3, EMMPRIN; 4, GDF15; 5, ICAM-1; 6, IGFBP-2; 7, MIF; 8, TFF3; 9, TfR. **B**. The relative pixel densities of each spot detected in the cytokine array analyzed by ImageJ. The ratio of the number of treated cells to the number of un-treated cells for each treatment is shown in the graph. NT, not treated. n=2. **C**. Quantitative RT-PCR of MCF7 cells treated with GDF15 (200 ng/mL) in the presence or absence of U0126 (5 μM). Transcripts were collected at the indicated time. NT, not treated. n=3. ***P < 0.001. **D**. Quantitative RT-PCR of T47D cells treated with GDF15 (200 ng/mL). Transcripts were collected at the indicated time. NT, not treated. n=3. ***P < 0.001. **E**. Quantitative RT-PCR analysis of MCF7 cells treated with GDF15 (200 ng/mL) and the indicated concentration of the anti-GDF15 antibody. Transcripts were collected at the indicated time. NT, not treated. n=3. **P < 0.01. **F**. Estimated model of the GDF15-ERK1/2-GDF15 circuit in the promortion of tumor sphere formation.

Because GDF15 induces activation of ERK1/2 over a prolonged period of time, we speculated that GDF15 induces the production of some cytokines or growth factors that lead to activation of ERK1/2. We used a cytokine array to identify cytokines or growth factors that are specifically induced by stimulation with GDF15, but not by TGFβ. Surprisingly, we found that GDF15, but not TGFβ, specifically induced its own expression even after 54 hours (Figure 4A and 4B). We next examined whether the GDF15-induced production of GDF15 takes place at the transcription level. We found that transcripts of *GDF15* were greatly increased by stimulation with GDF15 after 24 and 50 hours (Figure 4C). Additionally, we showed that treatment with U0126 inhibited the levels of *GDF15* transcripts that had been increased using GDF15 stimulation. Similarly, in T47D, which is another cell line with the luminal subtype, we found that stimulation with GDF15 greatly increased the levels of *GDF15* transcripts after 24 and 50 hours (Figure 4D). We, then, treated the cells with a neutralizing antibody against GDF15. As expected, treatment with an anti-GDF15 antibody dose-dependently decreased the expression levels of *GDF15* that had been increased using stimulation with GDF15 (Figure 4E). These results indicate that GDF15 induces its own expression at the transcription levels and then produces GDF15 protein. Thus, GDF15 may trigger the formation of GDF15-ERK1/2-GDF15 circuits and the circuits are maintained even after the effects of the addition of exogenous GDF15 are lost over time (Figure 4F).

### Expression levels of GDF15 are heterogeneous among cancer cells in the MCF7 cell line and in human breast cancer tissues

**Figure 5:**
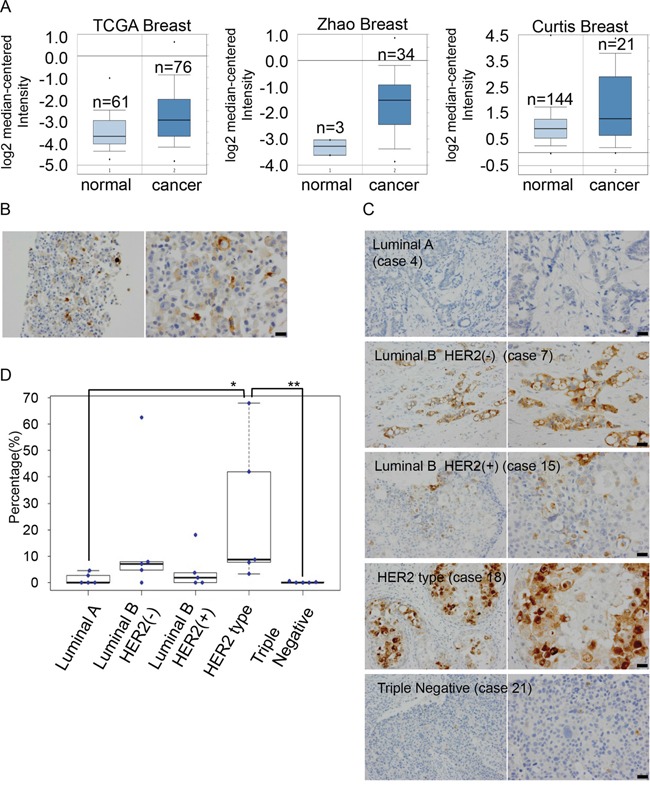
Expression levels of GDF15 are heterogeneous among cancer cells in MCF7 cells and human breast cancer tissues **A**. Analysis of the expression levels of *GDF15* transcripts in Oncomine database. **B**. Immunohistochemical staining of GDF15 in MCF7 cells in paraffin blocks using an anti-GDF15 antibody. Left, original magnification 200x. Right, scale bar: 20 μm. **C**. Immunohistochemical staining of GDF15 in various subtypes of breast cancer tissues. Left, original magnification 400x. Right, scale bar: 20 μm. **D**. Box plots of GDF15 expression among 25 clinical breast cancer tissues that include 5 cases in each subtype. **P < 0.01, *P < 0.05.

To compare the expression levels of *GDF15* in normal breast tissues and breast cancer tissues, we analyzed the Oncomine database (https://www.oncomine.org/resource/login.html). Expression levels of GDF15 are significantly higher in breast cancer tissues than in normal breast tissues (Figure 5A). To analyze cancer cells where the GDF15 autocrine/paracrine circuits are potentially active, even in the absence of exogenous GDF15 stimulation, we first examined expression of GDF15 in MCF7 cells by immunocytochemistry (Figure 5B). We observed that GDF15 was expressed in the cytoplasm; furthermore, the expression levels of GDF15 were very heterogeneous and were strong only in a few cells. This is consistent with the fact that cell populations even in a cell line are heterogeneous [30].

We next examined the expression of GDF15 in various subtypes of breast cancer tissues (Supplementary Table 2). GDF15 was expressed in the cytoplasm and expression levels of GDF15 were very low in luminal A type or triple negative type (Figure 5C, 5D and Supplementary Table 2) tissues. There were no cases where GDF15 positive cells were more than 5% (0/5 cases in luminal A type and 0/5 cases in triple negative type). On the other hand, expression levels of GDF15 varied greatly in luminal B type or HER2-positive type. GDF15 was expressed in more than 5% cell population in several cases (2/5 cases in luminal B type, 1/5 case in luminal B plus HER2 positive type and 4/5 cases in only HER2 positive type [estrogen receptor or progesterone receptor-negative]) and in more than 15% cell population in a few cases (1/5 case in luminal B type, 1/5 case in luminal B plus HER2 positive type and 2/5 cases in only HER2 positive type). Moreover, expression levels of GDF15 were heterogeneous among cancer cells. These results suggest that GDF15 circuits are active in a various amount of cell population.

## DISCUSSION

In this study, we hypothesize that GDF15 maintains cancer stem-like cells in an autocrine/paracrine manner; GDF15-activated ERK1/2 seems to induce *GDF15* at transcription levels, resulting in continuous production of GDF15 protein. Once the GDF15-ERK1/2-GDF15 circuits are formed, cancer stem-like cells dependent on the GDF15 circuits can be maintained in cancer tissues and would not be eliminated easily by conventional chemotherapy. To eradicate tumors, it is important to target GDF15 circuits to eliminate cancer stem-like cells. Targeting therapy, using neutralizing anti-GDF15 antibodies, would be a good strategy for this purpose.

It is important to select appropriate patients who should be treated by targeting the GDF15 circuits. We showed that expression levels of GDF15 are heterogeneous among cancer cells, even in the same breast cancer tissues. In ∼1/3 cases, GDF15^high^ cells are a very minor population, less than 5%. Because we examined a small number of cases within each subtype of breast cancer, it is possible that the extremely high expression of GDF15 in human breast cancer tissues will be observed regardless of the subtype. Consistent with the fact that CSCs are thought to be a minor population of cancer cells, partly due to relatively low proliferating activity [3], it is possible that GDF15-positive cells represent cancer stem-like cells. On the other hand, there are more than 15% GDF15^high^ cells in a few cases among luminal B type- or HER2-positive type- breast cancer tissues. We hypothesize that GDF15^high^ cancer stem-like cells gain high proliferating activity by using the highly active GDF15 circuits in these cancer tissues. If so, they would be appropriate cases for treatments that target GDF15 circuits. To prove our hypothesis, we will need to analyze the molecular mechanisms in greater detail, using HER2-positive or basal-type breast cancer cells.

Exogenously added GDF15 induced tumor sphere formation in primary cancer cells derived from luminal A type breast cancer tissues, though endogenous expression levels of GDF15 were very low. It is possible that a large amount of exogenous GDF15 triggers formation of the GDF15 circuits in cancer cells where GDF15 circuits are not active.

The signaling pathway of GDF15 is poorly understood and the “canonical” pathway of GDF15 signaling is unknown. Although GDF15 is a member of the TGF-β family, the GDF15-stimulated phosphorylation of Smad2 was modest compared to the strong phosphorylation of Smad2 by TGF-β. Instead, GDF15-stimulated phosphorylation of ERK1/2 was strongly and continuously observed even in a delayed time course. Several studies have also reported transient activation of ERK1/2 in HER2-overexpressing breast cancer cells by the transactivation of the ErbB2 (HER2) via GDF15-triggered TGFβ receptor-Src activation [27] [28] [31]. The activation of ERK1/2 increases rapidly, reaches its peak after ∼5∼10 min, and is downregulated to basal levels after ∼60 min. We showed that a ∼6-hour treatment with GDF15 induces a prolonged activation of ERK1/2 in MCF7 cells that do not overexpress HER family members. The activity of ERK1/2 declined after ∼24 hours but increased again after ∼50 hours. Prolonged activation of ERK1/2, using stimulation with GDF15, has not been reported previously. The reduction in the activity of ERK1/2 at 24 hours is likely caused by the time required to accumulate the levels of GDF15 produced by stimulation with GDF15; the amount of GDF15 at 24 hours is not yet sufficient to fully stimulate ERK1/2. It appears that the amount of GDF15, produced by stimulation with GDF15, is sufficient to stimulate ERK1/2 after ∼50 hours. Whether or not HER family members are involved in activation of ERK1/2, after a prolonged stimulation with GDF15, will be the subject of our future study. To clarify this issue, we need to perform more experiments. We would like to examine this in our future study.

It is still unknown how the GDF15-ERK1/2-GDF15 circuits induce tumor sphere formation. We previously showed that FGF stimulates sustained activation of ERK1/2 and induces expression of Hes1, a transcription factor regulating stemness [32]. Then, increased expression of Hes1 contributes to formation of neurospheres in mouse neural stem/progenitor cells. It is possible that this pathway is also activated in breast cancer stem-like cells.

There is a recent report suggesting that GDF15 may function in CSCs from multiple myeloma, a relatively rare subtype of hematological malignancy [33]. Our study supports the notion that GDF15 plays roles in CSCs not only in breast cancer but also in many other solid tumors.

## MATERIALS AND METHODS

### Cell lines and cell culture

MCF7, T47D, BT20, MDA-MB-436, and BT474 cell lines were purchased from the American Type Culture Collection (ATCC) and cultured in RPMI 1640 medium (Nacalai Tesque, Kyoto, Japan) supplemented with 10% fetal bovine serum (FBS) (JRH Biosciences, Kansas, MO) and 1% penicillin-streptomycin (Nacalai Tesque). All the cells were maintained routinely at 37 °C in humidified conditions with 5% CO_2_.

### Primary cell culture and tumor sphere formation assay

Clinical breast carcinoma samples used for culturing were provided from The University of Tokyo Hospital, Minamimachida Hospital, and Showa General Hospital (Supplementary Table 1). They were processed into primary tumor cells as previously described [11] and cultured in EpiCult™-B (Stemcell Technologies, Vancouver, Canada) with 1% penicillin-streptomycin. We previously confirmed that patient-derived tumor cells plated at 5,000 cells/mL yield tumor spheres that are clonally derived from single cells. We analyzed primary breast cancer cell cultures before the tenth passage. The sphere formation assay was performed as previously described [11]. Briefly, cells were plated as single cells on ultralow attachment 24-well plates (1,000-2,500 cells/well of cell lines, included MCF7 cells, 5,000 cells/well of patient-derived tumor cells). Spheres were grown in SCM containing 20 ng/mL epidermal growth factor (EGF) (Millipore, Darmstadt, Germany), 20 ng/mL basic fibroblast growth factor (bFGF) (PeproTech, New Jersey, NJ), B27 (Thermo Fisher Scientific, Waltham, MA) and heparin (Stem Cell Technologies) or in DMEM/F-12 medium supplemented with 200 ng/mL recombinant human GDF15 (R&D Systems Inc., Minneapolice, MN, cat.#957-GD) or 200 ng/mL recombinant human TGF-β (R&D Systems Inc., cat.#240-B). Cells were treated with or without SB431542 (Sellckchem, Houston, TX, cat.#S1067) or U0126 (Cell Signaling, Danvers, MA, cat.#9903) in the experiments using the inhibitors. Cells were treated with ligands and inhibitors every two days. Spheres >75 μm in diameter were counted after 4-7 days. Sphere-forming efficiency was calculated as the ratio of the number of spheres formed to the number of cells originally plated.

### MTT assays

A total of 1.0×10^3^ cells were seeded to 96-well plate and incubated at 37°C in humidified conditions with 5% CO_2_, then serum-starved overnight. Cell Titer 96® Non-Radioactive Cell Proliferation Assay (Promega, Madison, WI) was used to run the assay, according to the manufacturer's protocol. Cells were treated with GDF15 (200 ng/mL) or TGFβ1 (200 ng/mL) and further incubated for 3-5 days. The data was collected at the indicated time points.

### Cell cycle analysis

Cells were seeded in 35-mm adherent plates and cultured in the RPMI 1640 medium (Nacalai Tesque). Cells were starved overnight before treatment with 200 ng/mL of recombinant human GDF15. U0126 was added 1 hour before treatment with GDF15. Cells were harvested 50 hours after treatment with GDF15. Cells were washed twice with phosphate buffered saline (PBS; Nacalai Tesque) and fixed with 10 ml of cold 70 % ethanol (Wako, Osaka, Japan) overnight at -20°C. Then, the cells were washed twice, as described above, and incubated for 30 minutes at 37°C with 0.5 mL of RNase A (Nacalai Tesque) at a final concentration 0.25 mg/mL. Cells were incubated for another 30 minutes at 4°C with 5 μL of propidium iodide staining solution (BD Biosciences, San Jose, CA) and then analyzed using flow cytometry.

### Quantitative real time PCR (RT-PCR) analysis

Cells were seeded in adherent plates and cultured in the RPMI 1640 medium. Cells were starved overnight before treatment with 200 ng/mL of recombinant human GDF15. U0126 was added 1 hour before treatment with GDF15. To neutralize GDF15, anti-GDF15 (R&D systems Inc., cat#AF957) was added 24 hours after treatment with GDF15, without changing the medium. Total RNA was prepared at the indicated time using the RNeasy Micro kit (Qiagen, Hilden, Germany) and transcribed into cDNA using a High Capacity cDNA Reverse Transcription kit (Applied Biosystems, Carlsbad, CA). Quantitative RT-PCR was performed using Taqman probes (Applied Biosystems), according to the manufacturer's recommendations.

### Western blot analysis

Western blotting was performed using standard procedures as described [12]. Briefly, cells were seeded in adherent plates and cultured in the RPMI 1640 medium. Cells were starved overnight before treatment with 200 ng/mL of recombinant human GDF15 or 200 ng/mL of recombinant human TGF-β. SB431542 or U0126 was added 1 hour before treatment with GDF15 or TGF-β. Proteins were collected at the indicated times using RIPA buffer (Thermo Fisher Scientific) supplemented with a phosphatase inhibitor (Nacalai Tesque) and an EDTA-free protease inhibitor cocktail (Nacalai Tesque). Proteins were quantified using ProStain Protein Quantification Kit (Active Motif, Carlsbad, CA, cat. #15001). Anti-Smad2 (cat. #5339), p-Smad2 (cat. #3108), ERK1/2 (cat. #9122), and p-ERK1/2 (cat. #9101) antibodies were purchased from Cell Signaling Technology. Proteins were detected with horseradish peroxidase-conjugated anti-mouse or anti-rabbit antibodies (GE Healthcare, Little Chalfont, United Kingdom). The LAS 4000 mini (Fujifilm, Tokyo, Japan) was used to detect the blots.

### Cytokine array

Cells were seeded into adherent plates and cultured in the RPMI 1640 medium. The cells were starved overnight before treatment with 200 ng/mL of recombinant human GDF15 or 200 ng/mL of recombinant human TGF-β. Lysis Buffer 17 (R&D Systems) supplemented with 10 μg/mL Aprotinin (Sigma), 10 μg/mL Leupeptin (Tocris, Bristol, United Kingdom), and 10 μg/mL Pepstatin (Tocris) was used to collect the cell lysates. The cells lysates were used as the cytokine array samples. Cytokine array was performed using the Human XL Cytokine Array Kit (Proteome Profiler Array; R&D Systems), a kit for detecting 102 cytokines, according to the manufacturer's protocol. The LAS 4000 mini (Fujifilm) was used to detect the spots.

### Immunohistochemistry and scoring of positive cells

To generate a cell block of MCF7 cells, cells were cultured in a dish, washed with PBS, and collected by scraping. After centrifugation, the cells were fixed using Mildform (Wako, Kyoto, Japan) and embedded in paraffin. Sections (4-μm thick) were deparaffinized and incubated with an anti-GDF15 antibody (polyclonal, 1:200, Atlas Antibodies, Stockholm, Sweden). Human breast carcinoma specimens were obtained from the Kanazawa Medical University Hospital (Supplementary Table 2). Sections 4 μm thick were cut from 10% neutral buffered formalin-fixed paraffin-embedded blocks of each tumor, deparaffinized, and stained using a Bond-Max autostainer (Leica Microsystems, Tokyo, Japan), according to the manufacturer's protocol, with appropriate positive and negative controls. Antigen retrieval was carried out at pH 9 with an Epitope Retrieval 2 solution (Leica) for 20 minutes at 100 °C. Slides were then incubated for 15 minutes at room temperature with an anti-GDF15 antibody (polyclonal, 1:200, Atlas Antibodies, Stockholm, Sweden). A Leica Bond-Max avidin-biotin-free polymer system was used in the detection following the company's recommended procedure. Diaminobenzidine tetrahydrochloride was used as the chromogen. Slides were then counterstained with hematoxylin.

The immunostained sections were initially scanned at low power to determine the hot spots of the case by light microscopy (ECLIPSE 80i; Nikon, Tokyo, Japan). Images of the spots in 200x microscopic fields were captured by a digital camera (DP25, Olympus, Tokyo, Japan). The number of tumor cells stained with the anti-GDF15 antibody was determined by a pathologist (H. M.) as the percentage of 1000 consecutive tumor cells counted manually in a blind fashion.

### Statistical analysis

A student's unpaired *t*-test was used to compare differences between two samples. For tumor sphere-forming efficiency, tumor volume, and tumor weight, a paired two-tailed t-test was used. For comparison of the immunohistochemical results of GDF15 staining between breast cancer types, Man-Whitney U test was used. Values are presented as mean ± SD. Values of p < 0.01-0.05 (*), p < 0.001-0.01 (**), or p < 0.001(***) were considered significant.

### Study approval

This study was approved by the institutional review boards of the Institute of Medical Science, University of Tokyo, the University of Tokyo Hospital, Minamimachida Hospital, Showa General Hospital, Kanazawa Medical University, and Kanazawa University. Written informed consent was received from all the participants prior to inclusion in the study.

## SUPPLEMENTARY MATERIALS TABLES


