# Supplementary material for: An autocrine/paracrine circuit of growth differentiation factor (GDF) 15 has a role for maintenance of breast cancer stem-like cells
Source: Oncotarget. 2017 Feb 11;8(15):24869–81. doi: 10.18632/oncotarget.15276 (PMC5421895; doi:10.18632/oncotarget.15276)
Supplement: Supplementary file 1 [file oncotarget-08-24869-s001.pdf]

An autocrine/paracrine circuit of growth differentiation factor (GDF) 15 has a role for maintenance of breast cancer stem-like cells

SUPPLEMENTARY MATERIALS TABLES

Supplementary Table 1: Detailed information of the clinical samples used for sphere formation assay

Sample ID	Passages number	Subtype	Sphere formation
1	4	Luminal B HER2(+)	-
2	1	Luminal A	+
3	2	Luminal B HER2(-)	-
4	2	Luminal A	-
5	1	Luminal B HER2(-)	-
6	6	Luminal A	+
7	0	Luminal A	-
8	0	Luminal A	-

Supplementary Table 2: Detailed information of the clinical breast cancer tissue samples used for immunohistochemistry

Case ID	Specimen	Subtype	Nuclear grade	Lymph node status	Pathological stage	GDF15 positive cells(%)
1	Breast	Luminal A	1	-	I	0
2	Breast	Luminal A	1	+	IIA	0.1
3	Breast	Luminal A	2	+	IIA	4.7
4	Breast	Luminal A	1	-	I	0.1
5	Breast	Luminal A	1	-	I	2.7
6	Breast	Luminal B	1	-	I	0
7	Breast	Luminal B	1	+	IV	62.4
8	Breast	Luminal B	3	+	IIA	7.1
9	Breast	Luminal B	2	-	I	8
10	Breast	Luminal B	2	+	IIB	4.9
11	Breast	LuminalB HER2(+)	2	-	I	2
12	Lymph node	LuminalB HER2(+)	3	+	IIIA	3.8
13	Breast	LuminalB HER2(+)	1	-	I	0.1
14	Breast	LuminalB HER2(+)	3	-	IIA	0.2
15	Breast	LuminalB HER2(+)	1	-	I	18.1
16	Breast	HER2(+)	3	-	I	42
17	Soft tissue	HER2(+)	2	+	IV	7.8
18	Breast	HER2(+)	2	-	I	67.8
19	Breast	HER2(+)	2	+	IIIA	8.9
20	Breast	HER2(+)	3	-	IIA	3.4
21	Breast	Triple negative	3	-	IV	0.7
22	Skin	Triple negative	3	+	IV	0.1
23	Breast	Triple negative	3	-	IIA	0
24	Breast	Triple negative	3	-	I	0.2
25	Breast	Triple negative	3	-	I	0.3
